# Distribution and antimicrobial resistance profiles of bacterial species in stray cats, hospital-admitted cats, and veterinary staff in South Korea

**DOI:** 10.1186/s12917-020-02326-2

**Published:** 2020-04-09

**Authors:** Woo Kyung Jung, Sook Shin, Young Kyung Park, Suk-Kyung Lim, Dong-Chan Moon, Kun Taek Park, Yong Ho Park

**Affiliations:** 1 Department of Veterinary Microbiology, College of Veterinary Medicine and Research Institute for Veterinary Science, Seoul National University, Seoul, 08826 South Korea; 2 Bacterial Disease Division, Animal and Plant Quarantine Agency, 177 Hyeksin 8-ro, Gimcheon-si, Gyeongsangbuk-do 39660 South Korea; 3 Department of Biotechnology, Inje University, 197 Injero, Gimhae-si, Gyeongsangnam-do 50834 South Korea

**Keywords:** Cat, Antimicrobial resistance, *Staphylococcus* spp., *Enterobacteriaceae*, *Enterococcus* spp.

## Abstract

**Background:**

Antimicrobial resistance is becoming increasingly important in both human and veterinary medicine. According to the One Health concept, an important step is to monitor the resistance patterns of pathogenic bacteria. In this study, the antimicrobial susceptibility patterns and trends of bacteria isolated from stray cats, hospital-admitted cats, and veterinary staff in South Korea between 2017 and 2018 were investigated.

**Results:**

The minimum inhibitory concentrations of different antibiotics for *Staphylococcus* spp., *Enterobacteriaceae*, and *Enterococcus* spp. were determined to establish representatives of different antibiotic classes relevant for treatment or surveillance. For Coagulase-positive and Coagulase-negative Staphylococci, resistance to fluoroquinolones was below 13%, but resistance to ampicillin and penicillin was high (20–88%). A total of 9.5, 12.1, and 40.3% of staphylococcal isolates from stray cats, hospital-admitted cats, and veterinary staff, respectively, were confirmed to be *mecA* positive. For *Enterobacteriaceae*, resistance to carbapenems, fluoroquinolones, and 3rd generation cephalosporins was low (0–11.1%). The *Enterococcus* spp. isolates showed no resistance to vancomycin. The antimicrobial resistance rates of the *Staphylococcus* spp. and *Enterobacteriaceae* isolates from stray cats were usually lower than those of isolates from hospital-admitted cats and veterinary staff, but the *Enterococcus* spp. isolates revealed the opposite. Thus, the antimicrobial resistance varied across bacterial species according to the source from which they were isolated.

**Conclusions:**

Resistance to critically important compounds were low. However, the presence of antimicrobial resistance in cat isolates is of both public health and animal health concern.

## Background

Antimicrobials, including agents of importance to human medicine, are commonly used in companion animal-related veterinary practice [1, 2]. Transmission of antimicrobial resistance among bacteria from animals and veterinarians has been reported [3, 4]. Multidrug-resistant (MDR) *Staphylococcus intermedius/pseudintermedius* isolates are increasingly being reported to cause problems in small-animal practices [5–7]. According to Rusher et al., wounds and the ear canal are the most common sites for infections with methicillin-resistant *S. intermedius/pseudintermedius* [8]. Knowledge about drug resistance trends over time is important to ensure the long-term efficacy of antibacterial products, and data regarding antimicrobial susceptibility could help veterinarians select the most appropriate antibiotic for treatment purposes. Despite the importance of this aspect, standardized, ongoing surveys regarding antimicrobial resistance are barely available.

Companion animals such as dogs and cats not only share a common environment with humans, but are also administered drugs similar to those prescribed to humans. It has been postulated that companion animals may be an integral part of the transfer of drug resistance, owing to their close and direct contact with humans [9]. All kinds of companion animals (owned and stray animals) are involved in the transmission of drug resistance, even if the particular implication of each animal population has not yet been established clearly [10]. However, most of these concerns have been directed towards parasites of stray cats [11, 12]. Further, in Korea, a large number of studies have focused on dogs, rather than cats [13–15]. The rate of pet ownership is increasing globally, as animals enrich the lives of humans. In Korea, it has been estimated that over 29% (a total of 5.74 million) of households own a companion animal, with the estimated number of dogs and cats being 6.32 and 2.43 million, respectively [16]. Especially, the total number of companion cats is increasing rapidly, showing a growth of about 41% in 2017, compared to the statistic mentioned in a study from 2015 [16]. Stray cats that move freely within urban environments have also been shown to exist, although their population in Seoul has decreased from 200,000 in 2015 to 139,000 in 2017 due to the Trap-Neuter-Return program [17]. Therefore, the systematic control and prevention of drug resistance, through the implementation of a national antimicrobial resistance surveillance method, are greatly needed and should be applied in case of both cats and dogs. This study aimed to determine the antimicrobial susceptibility in the most common bacteria isolated from stray cats, hospital-admitted cats, and veterinary staff.

## Results

### Bacterial species

A total of 1829 *Staphylococcus* spp., *Enterobacteriaceae*, and *Enterococcus* spp. isolates were recovered (281 from stray cats, 978 from hospital-admitted cats, and 570 from veterinary staff). Table 1 shows the bacterial species isolated based on their origin (stray cats, hospital visiting cats, and veterinary staff). The most frequently isolated pathogens were *E. coli* in case of stray cats and hospital-admitted cats (*n* = 31, 11% and *n* = 161, 16.5%, respectively) and *S. epidermidis* in case of the veterinary staff (*n* = 89, 15.6%). Coagulase-negative Staphylococci (CNS) were isolated at a significantly higher (*P* < 0.05) rate from stray cats (64/78, 82.1%) than from hospital-admitted cats (230/350, 65.7%) and veterinary staff (160/294, 54.4%). Among the staphylococcal isolates, *S. felis* was isolated at a significantly higher frequency (*P* < 0.05) from stray cats and hospital-admitted cats (29/74, 39.2% and 60/264, 22.7%, respectively) than from veterinary staff (6/176, 3.4%). On the contrary, the isolation frequency of *S. epidermidis* from veterinary staff (89/176, 50.6%) was significantly higher (*P* < 0.05) than that from stray cats (6/74, 8.1%) and hospital-admitted cats (39/264, 14.8%).

**Table 1:** Bacterial isolates from 78 stray cats, 350 hospital-admitted cats, and 294 veterinary staff

Species identified		Stray cat (78)	Hospital-admitted cat (350)	Veterinary staff (294)
*Staphylococcus* spp.		74	264	176
Coagulase-positive Staphylococci (CPS)	*S. aureus*	5	29	10
	*S. intermedius*/*pseud intermedius*	3	5	5
	*S. hyicus*	2		1
Coagulase-negative Staphylococci (CNS)	*S. capitis*		7	4
	*S. caprae*	1	2	
	*S. chromogenes*	1	1	1
	*S. cohnii*	2	5	3
	*S. condimenti*		2	
	*S. epidermidis*	6	39	89
	*S. equorum*	1	8	1
	*S. felis*	29	60	6
	*S. gallinarum*			1
	*S. haemolyticus*		5	14
	*S. hominis*	3	17	4
	*S. lugdunensis*		1	5
	*S. nepalensis*	1	1	1
	*S. pettenkoferi*		6	1
	*S. saprophyticus*		14	9
	*S. schleiferi*	3	3	4
	*S. sciuri*	6	6	1
	*S. simulans*	9	26	6
	*S. warneri*	1	6	7
	*S. xylosus*	1	21	3
*Enterobacteriaceae*		39	189	50
*Citrobacter braakii*		1	1	
*Citrobacter* spp.		1	4	
*Enterobacter aerogenes*			4	12
*Enterobacter asburiae*			2	
*Enterobacter cloacae*		1	2	
*Enterobacter hormaechei*		1		
*Enterobacter kobei*				1
*Enterobacter ludwigii*			2	
*Enterobacter* spp.		1		
*Escherichia coli*		31	161	18
*Klebsiella pneumoniae*		2	4	2
*Klebsiella variicola*			4	
*Serratia liquefaciens*			1	
*Serratia marcescens*			1	17
*Serratia* spp.		1		
*Enterococcus* spp.		57	149	20
*Enterococcus avium*		3	5	
*Enterococcus casseliflavus*		2	2	2
*Enterococcus durans*				1
*Enterococcus faecalis*		25	74	9
*Enterococcus faecium*		13	38	5
*Enterococcus gallinarum*		4	7	
*Enterococcus hirae*		10	22	3
*Enterococcus saccharolyticus*			1	
Others		74	215	45
Total		281	978	570

### Antimicrobial susceptibility of *Staphylococcus* spp.

In case of the isolates of CNS from stray cats, the rates of resistance to ampicillin, penicillin, and oxacillin varied between 20 and 34%, and the rates of resistance to amox/clav, gentamicin, and tetracycline were 6–9%. Resistance to clindamycin, enrofloxacin, marbofloxacin, and chloramphenicol was absent or low (0–3%) (Table 3 and Fig. S1). For cephalexin, no CLSI breakpoints are available. The number of isolates of Coagulase-positive Staphylococci (CPS) from stray cats was too small; hence, the resistance rates of these isolates is not being presented.

More than half of the isolates of CPS from hospital-admitted cats showed resistance to ampicillin, penicillin, and amox/clav (59–82%) (Table 2 and Fig. S1). The rates of resistance to oxacillin, enrofloxacin, marbofloxacin, and tetracycline varied between 12 and 29%, and the rates of resistance to gentamicin, clindamycin, and chloramphenicol were lower (6–9%). For the isolates of CNS from hospital-admitted cats, the rates of resistance to ampicillin, penicillin, and oxacillin were between 35 and 60%, and the rates of resistance to amox/clav, gentamicin, and tetracycline were 11–24% (Table 3 and Fig. S1). The rates of resistance to clindamycin, enrofloxacin, marbofloxacin, and chloramphenicol were low (4–7%) (Table 3 and Fig. S1). In case of cephalexin, for which no breakpoints were available, the MIC distributions suggest the presence of acquired resistance for some isolates. The rates of resistance to ampicillin, penicillin, and tetracycline for isolates from hospital-admitted cats were significantly higher (*P* < 0.05) than those for the isolates from stray cats.

**Table 2:**
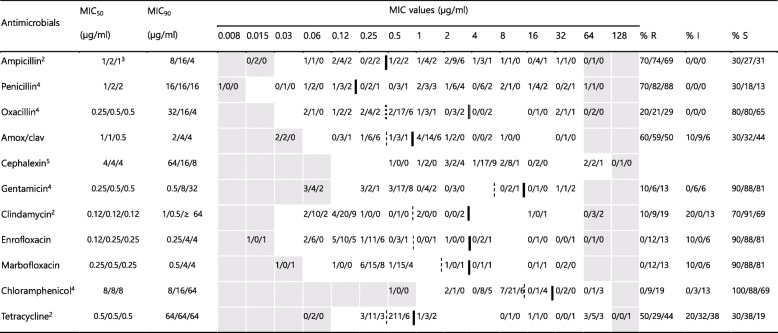
MICs of 11 antimicrobial agents against 60 Coagulase-positive Staphylococci^a^ isolated in this study

The dilution ranges tested are those contained in the white area. Values above this range indicate MIC values higher than the highest concentration within the range. Values below this range indicate MIC values lower than the lowest concentration within the range. Breakpoints are employed according to VET01S document. When available, susceptible and resistance breakpoints are indicated in vertical dotted and solid lines respectively. For antibiotics without intermediate zone, a single vertical solid line is indicated. For oxacillin, breakpoint of *S. aureus* is indicated in vertical doubled solid line and breakpoint of *S.* (*pseud*) *intermedius* and *S. hyicus* is in vertical doubled dotted line

MIC_50_, lowest concentration to inhibit 50% of bacteria; MIC_90_, lowest concentration to inhibit 90% of bacteria

*R* Resistant, *I* Intermediate, *S* Susceptible, *MIC* Minimum inhibitory concentration

^a^10 isolates from stray cats, 34 isolates from hospital-admitted cat, and 16 isolates from hospital staff

^b^Only CLSI breakpoint for dog isolates available

^c^Indicates isolates numbers from stray cat, hospital-admitted cat and hospital staff, respectively

^d^The breakpoints derived from human breakpoints used [18]

^e^No CLSI breakpoint available

**Table 3:**
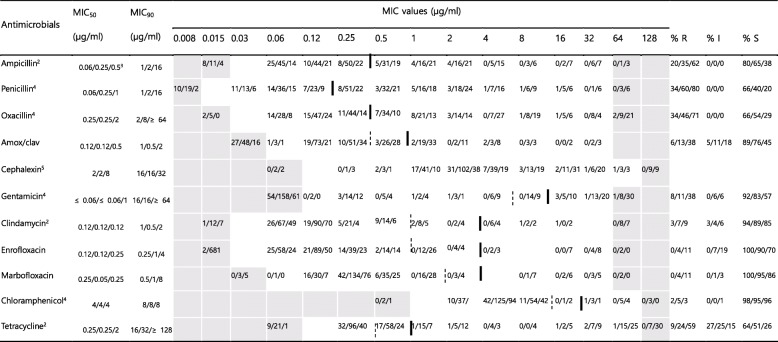
MICs of 11 antimicrobial agents against 454 Coagulase-negative Staphylococci^a^ isolated in this study

The dilution ranges tested are those contained in the white area. Values above this range indicate MIC values higher than the highest concentration within the range. Values below this range indicate MIC values lower than the lowest concentration within the range. Breakpoints are employed according to VET01S document. When available, susceptible and resistance breakpoints are indicated in vertical dotted and solid lines respectively. For antibiotics without intermediate zone, a single vertical solid line is indicated

MIC_50_, lowest concentration to inhibit 50% of bacteria; MIC_90_, lowest concentration to inhibit 90% of bacteria

*R* Resistant, *I* Intermediate, *S* Susceptible, *MIC* Minimum inhibitory concentration

^a^64 isolates from stray cats, 230 isolates from hospital-admitted cat, and 160 isolates from hospital staff

^b^Only CLSI breakpoint for dog isolates available

^c^Indicates isolates numbers from stray cat, hospital-admitted cat and hospital staff, respectively

^d^The breakpoints derived from human breakpoints used [18]

^e^No CLSI breakpoint available

In case of the CNS isolates from the veterinary staff, the rates of resistance to ampicillin, penicillin, oxacillin, and tetracycline varied between 59 and 80%, and the rates of resistance to amox/clav and gentamicin were 38%. The rates of resistance to clindamycin, enrofloxacin, marbofloxacin, and chloramphenicol was low (3–11%) (Table 3 and Fig. S1). In case of cephalexin, for which no breakpoints were available, the MIC distributions suggest the presence of acquired resistance for some isolates, like the case for the isolates of CPS from the hospital-admitted cats. The rates of resistance to ampicillin, penicillin, oxacillin, amox/clav, gentamicin, enrofloxacin, marbofloxacin, and tetracycline for isolates from the veterinary staff were significantly higher (*P* < 0.05) than those for the isolates from stray cats or hospital-admitted cats.

### Antimicrobial susceptibility of *Enterobacteriaceae*

For *Enterobacteriaceae* isolates from stray cats, resistance to ceftiofur, ceftriaxone, gentamicin, imipenem, meropenem, enrofloxacin, marbofloxacin, and tetracycline was absent or low (0–5%). The rate of resistance to cefoxitin was shown to be 13%. The rates of resistance to ampicillin, amox/clav, and cephalothin varied between 72 and 100% (Table 4 and Fig. S1).

**Table 4:**
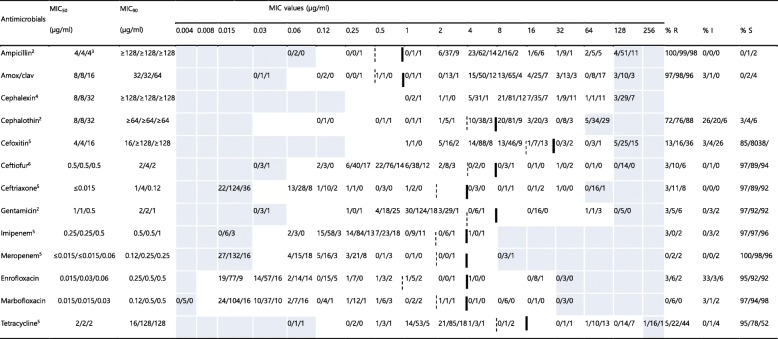
MICs of 13 antimicrobial agents against 278 *Enterobacteriaceae*^a^ isolated in this study

The dilution ranges tested are those contained in the white area. Values above this range indicate MIC values higher than the highest concentration within the range. Values below this range indicate MIC values lower than the lowest concentration within the range. Breakpoints are employed according to VET01S document. When available, susceptible and resistance breakpoints are indicated in vertical dotted and solid lines respectively

MIC_50_, lowest concentration to inhibit 50% of bacteria; MIC_90_, lowest concentration to inhibit 90% of bacteria

*R* Resistant, *I* Intermediate, *S* Susceptible, *MIC* Minimum inhibitory concentration

^a^39 isolates from stray cats, 189 isolates from hospital-admitted cat, and 50 isolates from hospital staff

^b^Only CLSI breakpoint for dog isolates available

^c^Indicates isolates numbers from stray cat, hospital-admitted cat, and hospital staff, respectively

^d^No CLSI breakpoint available

^e^The breakpoints derived from human breakpoints used [18]

^f^Only CLSI breakpoint for cattle isolates available

For *Enterobacteriaceae* isolates from hospital-admitted cats, resistance to gentamicin, imipenem, meropenem, enrofloxacin, and marbofloxacin was absent or low (0–6%). The rates of resistance to cefoxitin, ceftiofur, ceftriaxone, and tetracycline ranged from 10 to 22%. The rates of resistance to ampicillin, amox/clav, and cephalothin varied between 76 and 99% (Table 4 and Fig. S1). Tetracycline resistance in isolates from hospital-admitted cats was significantly higher (*P* < 0.05) than that in isolates from stray cats.

For *Enterobacteriaceae* isolates from veterinary staff, resistance to ceftiofur, ceftriaxone, gentamicin, imipenem, meropenem, enrofloxacin, and marbofloxacin was absent or low (0–8%). The rates of resistance to cefoxitin and tetracycline were between 36 and 44%, and were significantly higher (*P* < 0.05) than those of isolates from stray cats or hospital-admitted cats. The rates of resistance to ampicillin, amox/clav, and cephalothin varied between 88 and 98% (Table 4 and Fig. S1).

### Antimicrobial susceptibility of *Enterococcus* spp.

For *Enterococcus* spp. isolates from stray cats, rates of resistance to erythromycin, tetracycline, and minocycline were between 51 and 68%; however, resistance to ampicillin, vancomycin and high levels of resistance to streptomycin was absent or low (0–9%) (Table 5 and Fig. S1). The rates of resistance to penicillin, chloramphenicol, and ciprofloxacin, and high levels of resistance to gentamicin were between 16 and 21%.

**Table 5:**
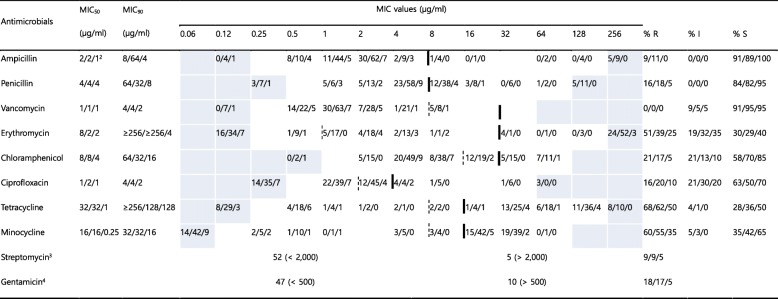
MICs of 10 antimicrobial agents against 226 *Enterococcus* spp.^a^ isolated in this study

The dilution ranges tested are those contained in the white area. Values above this range indicate MIC values higher than the highest concentration within the range. Values below this range indicate MIC values lower than the lowest concentration within the range. The breakpoints derived from human breakpoints used. When available, susceptible and resistance breakpoints are indicated in vertical dotted and solid lines respectively. For antibiotics without intermediate zone, a single vertical solid line is indicated

MIC_50_, lowest concentration to inhibit 50% of bacteria; MIC_90_, lowest concentration to inhibit 90% of bacteria

*R* Resistant, *I* Intermediate, *S* Susceptible, *MIC* Minimum inhibitory concentration

^a^57 isolates from stray cats, 149 isolates from hospital-admitted cat, and 20 isolates from hospital staff

^b^Indicates isolates numbers from stray cat, hospital-admitted cat, and hospital staff, respectively

^c^High-level resistance to streptomycin (2000 μg/ml)

^d^High-level resistance to gentamicin (500 μg/ml)

For *Enterococcus* spp. from hospital-admitted cats, rates of resistance to erythromycin, tetracycline, and minocycline were between 39 and 62%; however, resistance to vancomycin and high levels of resistance to streptomycin were absent or low (0–9%) (Table 5 and Fig. S1). The rates of resistance to ampicillin, penicillin, chloramphenicol, and ciprofloxacin, and high levels of resistance to gentamicin were between 11 and 20%.

For *Enterococcus* spp. from veterinary staff, rates of resistance to erythromycin, tetracycline, and minocycline were between 25 and 50%; however, resistance to ampicillin, penicillin, vancomycin, chloramphenicol, ciprofloxacin and high levels of resistance to both streptomycin and gentamicin were absent or low (0–10%) (Table 5 and Fig. S1).

### *mecA* gene detection

In total, 110 (21.4%) *Staphylococcus* spp. isolates harbored the *mecA* gene: 7 (9.5%) from stray cats, 32 (12.1%) from hospital-admitted cats, and 71 (40.3%) from veterinary staff. The rates of *mecA* among the staphylococcal isolates from the veterinary staff were higher than those among the staphylococcal isolates from the stray cats or hospital-admitted cats (*P* < 0.05). *S. epidermidis* was the most prevalent bacterium among the isolates from both the hospital-admitted cats and veterinary staff (15/46.9% and 53/74.7%, respectively) (data not shown). The oxacillin-resistance rates among the isolates of CNS were higher than those among the isolates of CPS from the hospital-admitted cats and veterinary staff (*P* < 0.05). Generally, the susceptibility ranges of the *mecA*-containing staphylococci isolated from the stray cats, hospital-admitted cats, and veterinary staff were similar. The rates of resistance to ampicillin and penicillin were between 84.4 and 98.6%, and the rates of resistance to amox/clav, gentamicin, and tetracycline were between 28.6 and 88.7%. Resistance to clindamycin, enrofloxacin, marbofloxacin, and chloramphenicol was absent or low (0–15.5%) (Table 6).

**Table 6:** MICs of 11 antimicrobial agents against 110 *mecA-*positive staphylococci isolated in this study

Antimicrobials	stray cats (7)					hospital-admitted cats (32)					veterinary staff (71)				
	MIC_50_ (μg/ml)	MIC_90_ (μg/ml)	% R	% I	% S	MIC_50_ (μg/ml)	MIC_90_ (μg/ml)	% R	% I	% S	MIC_50_ (μg/ml)	MIC_90_ (μg/ml)	% R	% I	% S
Ampicillin^a^	2	32	85.7	0	14.3	2	32	84.4	0	15.6	2	16	88.7	0	11.3
Penicillin^b^	1	≥ 64	85.7	0	14.3	2	16	96.9	0	3.1	2	32	98.6	0	1.4
Oxacillin^b^	2	≥ 64	100	0	0	16	≥ 64	100	0	0	4	≥ 64	100	0	0
Amox/clav	1	8	57.1	28.6	14.3	1	8	53.1	37.5	9.4	1	4	66.2	19.7	14.1
Cephalexin^c^	16	64				16	64				16	64			
Gentamicin^b^	0.25	32	28.6	0	71.4	8	≥ 64	40.6	18.8	40.6	32	≥ 64	60.6	5.6	33.8
Clindamycin^a^	0.5	16	14.3	28.6	57.1	0.12	4	12.5	0	87.5	0.12	8	15.5	5.6	78.9
Enrofloxacin	0.25	2	0	14.3	85.7	0.25	4	12.5	15.6	71.9	0.5	16	14.1	32.4	53.5
Marbofloxacin	0.25	2	0	14.3	85.7	0.5	16	12.5	0	87.5	0.5	8	14.1	5.6	80.3
Chloramphenicol^b^	4	32	14.3	0	85.7	4	8	3.1	0	96.9	4	8	2.8	1.4	95.8
Tetracycline^a^	32	64	57.1	28.6	14.3	1	64	53.1	25	21.9	64	≥ 128	88.7	5.6	5.6

Breakpoints are employed according to VET01S document

MIC_50_, lowest concentration to inhibit 50% of bacteria; MIC_90_, lowest concentration to inhibit 90% of bacteria

*R* Resistant, *I* Intermediate, *S* Susceptible, *MIC* Minimum inhibitory concentration

^a^Only CLSI breakpoint for dog isolates available

^b^The breakpoints derived from human breakpoints used [18]

^c^No CLSI breakpoint available

### Extended spectrum β-lactamases (ESBL) and carbapenemase detection

Among the 278 *Enterobacteriaceae* isolates, 1 *K. pneumoniae* isolate from a stray cat and 1 *K. pneumoniae*, 1 *Enterobacter asburiae*, 1 *Enterobacter cloacae*, and 11 *E. coli* isolates from hospital-admitted cats fulfilled the selection criteria for being termed as ESBL-producing bacteria. Among them, five *E. coli* isolates from hospital-admitted cats were confirmed to possess both *bla*_TEM_ and *bla*_CTX-M_, while one *K. pneumoniae* isolate from a stray cat and one *K. pneumoniae* isolate from a hospital-admitted cat possessed only *bla*_TEM_. None of the isolates possessed *bla*_SHV_. A total of eight isolates that satisfied the ESBL-producing bacteria selection criteria were negative for all resistance genes tested.

None of the *Enterobacteriaceae* isolates that were not susceptible to imipenem or meropenem showed enhanced growth in the modified Hodge test.

### Phenotypic resistance patterns derived from human clinical breakpoints

Overall, 15% (9/60) of the isolates of CPS, 25.3% (115/454) of the isolates of CNS, 48.6% (135/278) of the *Enterobacteriaceae* isolates, and 27.9% (63/226) of the *Enterococcus* spp. isolates were susceptible to all antimicrobial agents tested. Additionally, 30% (18/60) of the isolates of CPS, 33% (150/454) of the isolates of CNS, 25.5% (71/278) of the *Enterobacteriaceae* isolates, and 29.2% (66/226) of the *Enterococcus* spp. isolates showed MDR. The MDR rates of the isolates of CNS obtained from the veterinary staff (92/160, 57.5%) were significantly higher (*P* < 0.05) than those of the isolates of CNS obtained from the stray cats (7/64, 10.9%) or hospital-admitted cats (51/230, 22.2%) (Table 7). The MDR rates of the isolates of CNS obtained from the hospital-admitted cats were also significantly higher (*P* < 0.05) than those of the isolates of CNS obtained from the stray cats. The MDR rates of *Enterobacteriaceae* isolates obtained from veterinary staff (22/50, 44%) were significantly higher (*P* < 0.05) than those of *Enterobacteriaceae* isolates obtained from the stray cats (4/39, 10.3%) or hospital-admitted cats (45/189, 23.8%) (Table 8). On the contrary, the MDR rates of *Enterococcus* spp. isolates obtained from the veterinary staff (1/20, 5.0%) were significantly lower (*P* < 0.05) than those of *Enterococcus* spp. isolates obtained from the stray cats (18/57, 31.6%) or hospital-admitted cats (47/149, 31.5%) (Table 9).

**Table 7:** The most prevalent resistance profile per antimicrobial category found in Coagulase-negative Staphylococci isolated in this study based on CLSI human clinical breakpoint data

No. antimicrobial category	No. of isolates (%)	Resistance pattern (no. of isolates)		
		stray cats (*n* = 64)	hospital-admitted cats (*n* = 230)	veterinary staff (*n* = 160)
All susceptible	115 (25.3)	31	64	20
1	109 (24.0)	OXA (11)	BLA (37)	BLA (18)
2	80 (17.6)	BLA-OXA (4)	BLA-OXA (38)	BLA-OXA (15)
3	54 (11.9)	AMG-BLA-OXA (1)	BLA-BLI-OXA (14)	BLA-BLI-OXA (9)
4	38 (8.4)	BLA-BLI-OXA-TET (2)	AMG-BLA-OXA-TET (3)	AMG-BLA-OXA-TET (8)
5	39 (8.6)	–	AMG-BLA-BLI-OXA-TET (4)	AMG-BLA-BLI-OXA-TET (24)
6	18 (4.0)	AMG-BLA-BLI-OXA-LIN-TET (1)	AMG-BLA-BLI-OXA-FQN-TET (2)	AMG-BLA-BLI-OXA-FQN-TET (6)
7	3 (0.7)	–	AMG-BLA-BLI-OXA-LIN-PNC-TET (2)	AMG-BLA-BLI-OXA-FQN-LIN-TET (2)
Non-MDR	304 (67.0)	57 (89.1^a^)	179 (77.8^a^)	68 (42.5^a^)
MDR	150 (33.0)	7 (10.9^a^)	51 (22.2^a^)	92 (57.5^a^)

Antimicrobial categories included: aminoglycosides, AMG (gentamicin); β-lactam groups, BLA (ampicillin and penicillin); Oxacillin, OXA; β-lactam/β-lactamase inhibitor combination, BLI (amoxicillin/clavulanate); fluoroquinolones, FQN (enrofloxacin and marbofloxacin); lincosamide, LIN (clindamycin); phenicol, PNC (chloramphenicol); and tetracycline, TET

^a^Percentages

**Table 8:** The most prevalent resistance profile per antimicrobial category found in *Enterobacteriaceae* isolated in this study based on CLSI human clinical breakpoint data

No. antimicrobial category	No. of isolates (%)	Resistance pattern (no. of isolates)		
		stray cats (*n* = 39)	hospital-admitted cats (*n* = 189)	veterinary staff (*n* = 50)
All susceptible	135 (48.6)	29	99	7
1	32 (11.5)	BLA (2)	BLA (9)	BLA (3)
2	40 (14.4)	BLA-BLI (1)	BLA-TET (8)	GC-TET (9)
3	48 (17.3)	BLA-BLI-GC (1)	BLA-BLI-GC (11)	BLA-BLI-GC (9)
4	12 (4.3)	BLA-BLI-GC-CPM (1)	BLA-BLI-GC-3GC (3)	AMG-BLA-BLI-TET (1)
5	9 (3.2)	BLA-BLI-GC-3GC-TET (1)	BLA-BLI-GC-3GC-TET (4)	–
6	2 (0.7)	–	AMG-BLA- GC-3GC-FQN-TET (1)	–
Non-MDR	207 (74.5)	35 (89.7^a^)	144 (76.2^a^)	28 (56.0^a^)
MDR	71 (25.5)	4 (10.3^a^)	45 (23.8^a^)	22 (44.0^a^)

Antimicrobial categories included: aminoglycosides, AMG (gentamicin); β-lactam group, BLA (ampicillin); β-lactam/β-lactamase inhibitor combination, BLI (amoxicillin/clavulanate); 1st and 2nd generation cephalosporins, GC (cephalexin, cephalothin and cefoxitin); 3rd generation cephalosporins, 3GC (ceftiofur and ceftriaxone); fluoroquinolones, FQN (enrofloxacin and marbofloxacin); carbapenems, CPM (imipenem and meropenem); and tetracycline, TET

^a^Percentages

**Table 9:** The most prevalent resistance profile per antimicrobial category found in *Enterococcus* spp. isolated in this study based on CLSI human clinical breakpoint data

No. antimicrobial category	No. of isolates (%)	Resistance pattern (no. of isolates)		
		stray cats (*n* = 57)	hospital-admitted cats (*n* = 149)	veterinary staff (*n* = 20)
All susceptible	63 (27.9)	13	43	7
1	50 (22.1)	TET (8)	TET (23)	TET (6)
2	47 (20.8)	MAC-TET (10)	MAC-TET (16)	MAC-TET (3)
3	31 (13.7)	MAC-PNC-TET (5)	MAC-PNC-TET (8)	–
4	14 (6.2)	STM-MAC-PNC-TET (2)	AMG-MAC-PNC-TET (4)	AMG-MAC-PNC-TET (1)
5	12 (5.3)	AMG-BLA-FQN-MAC-TET (2)	AMG-BLA-FQN-MAC-TET (7)	–
6	6 (2.6)	AMG-STM-BLA-FQN-MAC-TET (2)	AMG-STM-BLA-FQN-MAC-TET (2)	–
7	3	–	AMG-STM-BLA-FQN-MAC-PNC-TET (3)	–
Non-MDR	160 (70.8)	39 (68.4^a^)	102 (68.5^a^)	19 (95.0^a^)
MDR	66 (29.2)	18 (31.6^a^)	47 (31.5^a^)	1 (5.0^a^)

Antimicrobial categories included: aminoglycosides except streptomycin, AMG (gentamicin); streptomycin, STM; β-lactam groups, BLA (ampicillin and penicillin); glycopeptide, GLP (vancomycin); fluoroquinolone, FQN (ciprofloxacin); macrolide, MAC (erythromycin); phenicol, PNC (chloramphenicol); and tetracycline, TET

^a^Percentages

The most common resistance profile among the CNS isolates was BLA-OXA, which was detected in 57 isolates of CNS, while the most common MDR profile was AMG-BLA-BLI-OXA-TET, which was observed in 4 isolates of CNS obtained from the hospital-admitted cats and 24 isolates of CNS obtained from the veterinary staff. Among the *Enterobacteriaceae* isolates, the most common resistance profile was BLA-BLI-GC, which was detected in 1 isolates of *Enterobacteriaceae* obtained from the stray cats, 11 isolates from hospital-admitted cats, and 9 isolates from the veterinary staff. In the *Enterococcus* spp. isolates, the most common resistance profile was MAC-TET, which was detected in 29 isolates of *Enterococcus* spp., while the most common MDR profile was MAC-PNC-TET, which was observed in 5 isolates of *Enterococcus* spp. obtained from the stray cats, and 8 isolates from the hospital-admitted cats.

## Discussion

In this study, we report the findings of the first nation-wide cross-sectional study of antimicrobial resistance in *Staphylococcus* spp., *Enterobacteriaceae*, and *Enterococcus* spp. isolates from stray cats, hospital-admitted cats, and veterinary staff in South Korea. The higher detection rate of CNS from stray cats than that among the isolates from hospital-admitted cats and veterinary staff may be caused by the poor, unhygienic surroundings of the stray cats. Among the samples from the veterinary staff, *S. epidermidis* was the major species isolated, followed by *E. coli* and *Serratia marcescens*. The most commonly identified staphylococcal species among isolates from both the stray cats and hospital-admitted cats was *S. felis*; this is in accordance with a previous German study, which showed that half of the *Staphylococcus* spp. isolates obtained were *S. felis*, but inconsistent with the results of studies in USA and South Africa, which have shown that *S. intermedius/pseudintermedius* and *S. epidermidis* were the representative staphylococci present in cats [19–21]. *S. felis* has been regarded as a normal commensal organism present on the skin, the conjunctival sac and eyelid margins, and in the saliva of normal healthy cats, as well as an etiological agent that causes skin infections such as pyoderma and otitis [22, 23]. On the other hand, the most frequently isolated staphylococcal species among samples from the veterinary staff was *S. epidermidis*, which is consistent with the results of other studies [24, 25]. This is presumably due to the different host specificities of the staphylococcal species [4].

Comparison of the results obtained in this study with those from international studies are difficult to interpret due to differences between the study design, drugs tested, breakpoint determination, and temporal or geographic variation. According to the ComPath project of Europe, *Pasteurella* spp. has been reported to be the major dermatological bacterial pathogen in cats, followed by *S. intermedius/pseudintermedius* and *S. aureus* [26]. For *S.* intermedius/pseudintermedius, the rates of resistance to penicillin, gentamicin, and chloramphenicol were 16–23%, while the rates of resistance to oxacillin, amox/clav, enrofloxacin, and marbofloxacin were 8–11%. In comparison, in case of *S. aureus*, high rates of resistance to penicillin (62%), as well as low rates of resistance to oxacillin, gentamicin, enrofloxacin, marbofloxacin, and chloramphenicol (0–7%) were found. In our study, the isolates of CPS which are including both of *S. intermedius/pseudintermedius* and *S. aureus* obtained from the stray cats and hospital-admitted cats showed higher rates of resistance to penicillin, oxacillin, and amox/clav than those observed in case of the isolates analyzed in the ComPath study. However, we observed slightly lower levels of resistance to enrofloxacin and marbofloxacin among isolates of stray cats than those observed in the ComPath study.

The isolates of CNS showed higher rates of resistance to oxacillin than the isolates of CPS from the stray cats, hospital-admitted cats and veterinary staff. This has been previously reported in case of studies on both human and veterinary medicine [4, 27]. The isolates of CPS showed higher resistance than CNS to most of the tested antimicrobials except oxacillin; additionally, the isolates of CPS from stray cats showed significantly higher resistance to ampicillin, penicillin, amox/clav, and tetracycline, and those from hospital-admitted cats showed significantly higher resistance to ampicillin, penicillin, and amox/clav (*P* < 0.05).

In this study, 7 staphylococcal isolates from stray cats and 32 from hospital-admitted cats were identified to harbor the *mecA* gene; this corresponds to 9.5% of the isolates from the stray cats and 12.1% of the isolates from the hospital-admitted cats, respectively. These statistics are similar with those of the ComPath study, which showed that 10.3% of the isolates from cats harbored the *mecA* gene [26]. Among the 176 staphylococcal isolates from the veterinary staff, 71 (40.3%) possessed the *mecA* gene; this finding is similar to that of our previous study, which showed that the prevalence of the *mecA* gene in *S. intermedius/pseudintermedius* isolates from veterinary staff was 35% [28].

In an Australian study, 341 clinical *E. coli* isolates were collected from cats [29]. In this study, lower rates of resistance to cefoxitin, ceftiofur, ceftriaxone, gentamicin, imipenem, enrofloxacin, marbofloxacin, and tetracycline (0–10%) and higher rates of resistance to ampicillin, amox/clav, and cephalothin (15–100%) were observed. This is similar to the results of our study, except for the fact that in our study, the rates of resistance to ampicillin, cephalothin, cefoxitin and tetracycline were found to be higher.

Among the *Enterococcus* spp. isolates, the most prevalent species was *E. faecalis*, followed by *E. faecium*; this is in accordance with a German study, which showed that *E. faecalis* represented the vast majority of the *Enterococcus* spp. isolates (26 of 29 isolates) [21]. No *Enterococcus* spp. isolates were found to be resistant to vancomycin, a critically important drug whose use is strongly discouraged because it can be considered to be ‘reserved for the treatment of serious MRSA infections in humans’ [30].

*Enterobacteriaceae* and *Enterococcus* spp. were isolated from the samples of veterinary staff only at a low frequency; this may be caused by sampling bias, for e.g., bias due to the fact that only the skin and nasal swab samples were included.

Although limited data are available for comparison, *E. coli* isolates from hospitalized dogs have been found to be more resistant than those from stray dogs [13]. Similarly, the frequency of the resistance to every antimicrobial tested in this study was higher in case of the isolates of CNS from hospital-admitted cats than in case of those from stray cats; the isolates of CNS from hospital-admitted cats showed significantly higher levels of resistance to penicillin and tetracycline than those from stray cats (*P* < 0.05). The number of isolates of CPS was too small to perform a comparison. The resistance rates of *Enterobacteriaceae* and *Enterococcus* spp. isolates from stray cats and hospital-admitted cats were similar. The stray cats tested in this study may be assumed to be healthy cats, because the stray cats was available for the neuter surgery; and, it is true that the hospital-admitted cats had a greater chance of receiving medication. This could be the reason why the isolates of CNS from hospital-admitted cats showed higher levels of drug resistance. However, why there were no differences between the drug resistance rates of the *Enterobacteriaceae* and *Enterococcus* spp. isolates from stray cats and hospital-admitted cats was not understood; this finding has been considered to be a good indicator of the selection pressure caused by the use of antimicrobials [31].

Interestingly, the MDR rates of CNS and *Enterobacteriaceae* isolates from the veterinary staff were significantly higher than those of the isolates from both the stray cats and hospital-admitted cats (*P* < 0.05). These results support the hypothesis that veterinary staff may serve as possible reservoirs for the dissemination of multidrug resistance in veterinary hospitals. This highlights the importance of vigilance by veterinary staff. Veterinary staff may serve as carriers for the pathogens; in this study, it was observed that the veterinary staff from whom the bacterial isolates were obtained showed no clinical signs of diseases. The MDR rates of CNS and *Enterobacteriaceae* isolates from the stray cats were significantly lower than that of the isolates from the hospital-admitted cats (*P* < 0.05). This may reflect the fact that stray cats only had a rare opportunity to receive medication. However, it has been known that stray dogs show a higher rate of parasitic infection than housed dogs; this may be due to the scavenging habits of stray dogs, which make them more vulnerable to natural infection than housed dogs [10, 12].

The MDR rate of *Enterobacteriaceae* isolates from hospital-admitted cats was 23.8%, which was much higher than that seen in case of the Australian study (11.7% among *E. coli* isolates from cats) [29].

The ESBL detection rates among the isolates were as follows: 2.6% (1/39) of the isolates from stray cats, 3.2% (6/189) of the isolates from hospital-admitted cats, and 0% of the isolates from veterinary staffs. Among them, five *E. coli* isolates from hospital-admitted cats were confirmed to harbor both *bla*_TEM_ and *bla*_CTX-M_, while one *K. pneumoniae* isolate from a stray cat and one *K. pneumoniae* isolate from a hospital-admitted cat were observed to harbor only *bla*_TEM_. None of the isolates possessed *bla*_SHV_. Since 2000, CTX-M β-lactamases have been identified as the most widespread type of ESBLs, replacing classical TEM- and SHV-type ESBLs [32]. The ComPath study has reported a low prevalence of ESBL-producing bacteria in dogs (2.8%) and the absence of ESBL-producing bacteria in cats [26]. However, a previous Korean study has shown a higher prevalence of ESBL-producing isolates in dogs and cats (29.2% in dogs and 13.5% in cats) [33]. The differences in their prevalence may be caused by the use of an ESBL-selective agar (CHROMagar ESBL). AmpC β-lactamases were not investigated in this study; they need to be focused on in future studies.

## Conclusion

*Staphylococcus* spp., and *Enterobacteriaceae* isolates from stray cats and hospital-admitted cats in Korea showed higher resistance to most of the tested antimicrobials, compared to the isolates from cats in European countries and Australia. Additionally, some isolates showed resistance against antimicrobials that are regarded as critically important for use in humans, such as third-generation cephalosporins and quinolones. It is unclear if the higher resistance to certain kinds of antimicrobials was attributed to a result of misuse of antimicrobials in animal hospitals because the treatment history of cats was not collected in the current study. Nevertheless, considering the risk of cross-transmission of resistant bacteria between cats and humans, emergence and dissemination of antimicrobial resistance in cat will undoubtedly continue to be a challenge in veterinary medicine, from both patient health and public health standpoint. More organized surveillance is required to better understand the mechanism of antimicrobial resistance transmission between veterinary and human medicine, in accordance with the One Health perspective.

## Methods

### Sample collection

**Fig. 1:**
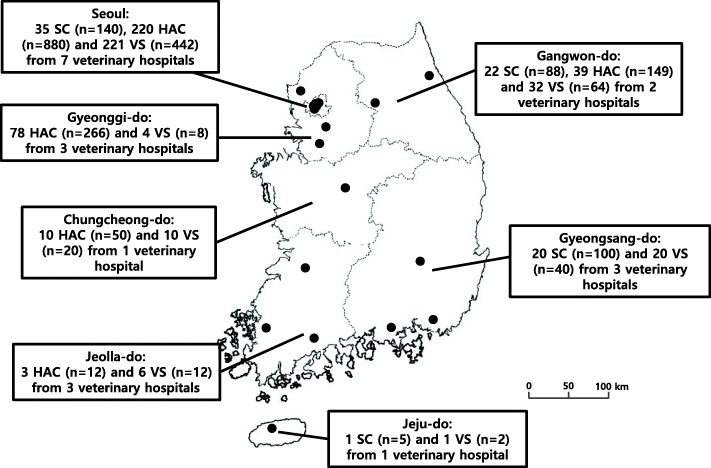
Geographical distribution of veterinary hospitals included on this study. The map shows physical locations of veterinary hospitals in which samples collected (closed circles) and the number of samples collected in each province. The number in parentheses indicates the total of swap samples collected from the indicated number of individual cats or staffs. SC, Stray cats; HAC, hospitals-admitted cats; VS, veterinary staffs. The map used in the figure was taken from the National Geographic Information Institute of Korea (https://www.ngii.go.kr/child/contend.do?sq=149)

A total of 2278 samples were obtained in convenience from 78 stray cats (*n* = 333), 350 hospital-admitted cats (*n* = 1357), and 294 veterinary hospital staff including veterinarians, veterinary technicians, and receptionists (*n* = 588) across 20 veterinary hospitals in Seoul, Gangwon-do, Gyeonggi-do, Chungcheong-do, Gyeongsang-do, Jeolla-do, and Jeju-do between 2017 and 2018 (Fig. 1). The stray cats were from the trap-neuter-return program. The captured cats were sent to the local veterinary hospital and the sampling was performed immediately after their arrival. Samples from the 333 stray cats and 1357 hospital-admitted cats were obtained from the anus (*n* = 425), horizontal ear canal (*n* = 416), nasal mucosa (*n* = 417), skin (*n* = 384), and urine (*n* = 48). All samples were collected by skilled veterinarians without anesthesia. The captured stray cats were released after the neutering. The hospital-admitted cats were proceeded to the appropriated treatment after sampling. Human samples were taken from the hand (palm and the skin between fingers; *n* = 294) and nasal cavity (*n* = 294). All samples from the cats and humans were obtained using BD BBL Culture Swabs (Becton-Dickinson, Sparks, MD, USA), placed on ice, and transported to our lab within 6 h of their collection.

### Bacterial isolation and identification

The swabs were directly plated on 5% defibrinated sheep blood agar (Hangang, Gunpo, South Korea) and incubated overnight at 37 °C for 24 h under aerobic and anaerobic conditions. All the colonies that showed different morphologies and hemolysis patterns were collected and subjected to Matrix-Assisted Laser Desorption Ionization-Time of Flight mass spectrometry (microflex LT/SH spectrometer Bruker, Bruker Daltonics, Bremen, Germany) for their identification. When multiple isolates of a same species were recovered from a given individual, only one isolate was selected for further analysis. *Staphylococcus* spp., *Enterobacteriaceae*, and *Enterococcus* spp. were most frequently isolated, so that these bacteria were mainly investigated in this study. Both *Staphylococcus intermedius* and *S. pseudintermedius* were designated as “*S.intermedius/pseudintermedius*” because of the difficulty associated with their differentiation.

### Antimicrobial susceptibility testing

Minimum inhibitory concentrations were determined using the standardized agar dilution methodology, as recommended by the Clinical and Laboratory Standards Institute (CLSI) guidelines [18, 34]. *Escherichia coli* ATCC 25922, *Staphylococcus aureus* ATCC 29213, and *Enterococcus faecalis* ATCC 29212 were included as quality control strains in the tests, as recommended by the CLSI guidelines. The antimicrobial susceptibility data of the isolates were obtained for the following antibiotics: gentamicin from the aminoglycoside except streptomycin (AMG); streptomycin (STM); ampicillin, and penicillin from the β-lactam (BLA) groups; oxacillin (OXA); amoxicillin/clavulanate (amox/clav, ratio 2:1) from the β-lactam/β-lactamase inhibitor combination (BLI); enrofloxacin, marbofloxacin, and ciprofloxacin from the fluoroquinolones (FQN); clindamycin from the lincosamide (LIN); chloramphenicol from the phenicol (PNC); cephalexin, cephalothin, and cefoxitin from the 1st and 2nd generation cephalosporins (GC); ceftiofur, and ceftriaxone from the 3rd generation cephalosporins (3GC); imipenem, and meropenem from the carbapenems (CPM); minocycline and tetracycline from the tetracyclines (TET); and vancomycin from the glycopeptide (GLP). The concentration ranges indicated in Tables 2, 3, 4 and 5. The isolates were defined as MDR if they showed resistance to at least one drug from three or more antimicrobial classes using human clinical breakpoints, as previously described [35].

### Data analyses

The MIC_50_ and MIC_90_ values of the tested antimicrobials were determined for *Staphylococcus* spp., *Enterobacteriaceae*, and *Enterococcus* spp., which were mainly isolated. Based on the MICs, the isolates were categorized as susceptible, intermediate, or resistant to the antibiotics for which CLSI veterinary breakpoints [34] or breakpoints based on human data [18] are available. The CLSI susceptibility and resistance breakpoints utilized are indicated in Tables 2, 3, 4 and 5.

The isolation and antimicrobial resistance rates of each bacterial species from different origins (stray cats, veterinary hospital admitted cats, and veterinary hospital staff) were analyzed and compared by Fisher’s exact test or Chi square test. All statistical analyses were performed using MedCalc statistical software ver. 11. 2. 1 (MEDCALC, Acacialaan, Belgium). In all tests, *P* values less than 0.05 were considered significant.

### Detection of the *mecA* gene

All *S. intermedius/pseudintermedius* and coagulase-negative Staphylococci (CNS) isolates for which the MICs of oxacillin were ≥ 0.5 μg/ml and all *S. aureus* isolates for which the MICs of oxacillin were ≥ 4 μg/ml [34] were screened by PCR for the presence of the *mecA* gene according to a method adapted from Oliverira D. C. et al. [36]. Concurrently, the *mecA*-positive strain *S. aureus* ATCC 43300 was used as a quality control organism.

### Detection of ESBLs and carbapenemase

The mechanisms underlying the resistance of *Enterobacteriaceae* towards β-lactams were further characterized. The presence of ESBLs was tested by a combined double-disc test using clavulanic acid and cephalosporin indicators (cefotaxime and cefrazidime), according to the CLSI guidelines [18]. *E. coli* ATCC 25922 and *Klebsiella pneumoniae* ATCC 700603 were used as a quality control strains for a combined double-disc test. Genotypic characterization of the ESBL-positive strains was achieved by performing PCR to detect the major groups of genes encoding β-lactamases, including *bla*_CTX-M_, *bla*_SHV_, and *bla*_TEM_ [37].

All *Enterobacteriaceae* strains that were not susceptible to imipenem or meropenem were tested by performing the modified Hodge test according to the CLSI guidelines [18]. This test represents a simple method for detecting carbapenemase-producing *Enterobacteriaceae*.

## Supplementary information

**Additional file 1 Figure S1.** Percentage resistance for antimicrobials against Coagulase-positive Staphylococci (A), Coagulase-negative Staphylococci (B), *Enterobacteriaceae* (C), and *Enterococcus* spp. (D) isolates from stray cats, hospital-admitted cats, and veterinary staff. The data sets labeled with different superscript letters (a, b, and c) are statistically different from each other (*P* < 0.05).

## Data Availability

The datasets used and/or analysed during the current study are available from the corresponding author on reasonable request.

## Abbreviations

MIC: Minimum inhibitory concentration
MDR: Multidrug-resistant
CLSI: Clinical and Laboratory Standards Institute
CNS: Coagulase-negative Staphylococci
CPS: Coagulase-positive Staphylococci
AMG: Aminoglycosides except streptomycin
STM: Streptomycin
BLA: β-lactam groups
OXA: Oxacillin
BLI: β-lactam/β-lactamase inhibitor combinations
FQN: Fluoroquinolones
LIN: Lincosamides
PNC: Phenicols
GC: 1st and 2nd generation cephalosporins
3GC: 3rd generation cephalosporins
CPM: Carbapenems
TET: Tetracyclines
GLP: Glycopeptides
amox/clav: Amoxicillin/clavulanate
ESBL: Extended spectrum β-lactamases
