# Supplementary material for: Distribution and antimicrobial resistance profiles of bacterial species in stray cats, hospital-admitted cats, and veterinary staff in South Korea
Source: BMC Vet Res. 2020 Apr 9;16:109. doi: 10.1186/s12917-020-02326-2 (PMC7147017; doi:10.1186/s12917-020-02326-2)
Supplement: Supplementary file 1 — Additional file 1 Figure S1. Percentage resistance for antimicrobials against Coagulase-positive Staphylococci (A), Coagulase-negative Staphylococci (B), Enterobacteriaceae (C), and Enterococcus spp. (D) isolates from stray cats, hospital-admitted cats, and veterinary staff. The data sets labeled with different superscript letters (a, b, and c) are statistically different from each other (P < 0.05). [file 12917_2020_2326_MOESM1_ESM.docx]

Additional file1: **Fig. S1** Percentage resistance for antimicrobials against Coagulase-positive Staphylococci (A), Coagulase-negative Staphylococci (B), Enterobacteriaceae (C), and Enterococcus spp. (D) isolates from stray cats, hospital-admitted cats, and veterinary staff. The data sets labeled with different superscript letters (a, b, and c) are statistically different from each other (P < 0.05).
